# Examining Flipped Classroom and Project-Based Learning Integration in Older Adult Health Education: A Mixed-Methods Study

**DOI:** 10.3390/nursrep15080267

**Published:** 2025-07-25

**Authors:** Fu-Chi Yang, Hsiao-Mei Chen

**Affiliations:** 1 Department of Healthcare Industry Technology Development and Management, College of Management, National Chin-Yi University of Technology, Taichung 411030, Taiwan; caring686868@gmail.com; 2 Liberal Education Center, College of General Education, National Chin-Yi University of Technology, Taichung 411030, Taiwan; 3 Department of Nursing, Chung Shan Medical University, Taichung 40201, Taiwan; 4 Department of Nursing, Chung Shan Medical Hospital, Taichung 40201, Taiwan

**Keywords:** flipped classroom, project-based learning, willingness to serve older adults, teamwork, nursing

## Abstract

**Background:** As population aging accelerates, the demand for professionals in older adult care continues to rise. Traditional teaching methods often fail to improve students’ willingness to serve older adults or foster teamwork. This study evaluated the effects of integrating a flipped classroom with project-based learning (PBL) and a hands-on clinical practicum into a health internship course. **Methods:** A mixed-methods design was adopted. Participants included 88 interdisciplinary university students enrolled in an 18-week, two-credit geriatric health internship course offered at a university in central Taiwan from August 2023 to July 2024. The course combined flipped classroom and PBL approaches, as well as clinical practicum activities. Data on willingness to serve older adults, teamwork skills, and learning outcomes were collected using structured questionnaires and analyzed with paired t-tests. **Results:** Significant improvements were found in willingness (from 68.93 to 73.15), teamwork (67.33 to 71.45), and learning outcomes (89.84 to 102.14) (*p* = 0.001). Qualitative findings further revealed increased empathy, improved teamwork, and enhanced ability to apply knowledge in real-world contexts. **Conclusions:** A teaching approach that integrates a flipped classroom, PBL, and a clinical practicum can effectively enhance students’ competencies in older adult care. Future research should explore long-term and cross-cultural impacts.

## 1. Introduction

Faced with aging societies, the demand for high-quality older adult care has significantly increased [1]. This demand underscores the urgency for educational systems to cultivate professionals capable of providing excellent care services for older adults [2,3]. However, traditional teaching methods primarily focus on theoretical knowledge while lacking opportunities for practical application and interactive learning. This limits students’ motivation to serve older adults and hinders the effective development of teamwork skills [4,5]. As a result, students frequently lack the necessary engagement and skills to meet the actual care needs of older adults. This challenge highlights the importance of developing innovative educational strategies to improve students’ learning outcomes while preparing them for future roles in older adult care [2,3].

Research suggests that innovative teaching methods such as flipped classrooms and project-based learning (PBL) can effectively enhance student engagement and learning outcomes [6]. The flipped classroom model reverses the traditional teaching process by allowing students to study theoretical content outside of classroom (e.g., digital materials and video lectures), enabling class time to focus on interactive discussions and practical activities [7]. This approach maximizes opportunities for deep learning and student interaction, helping learners to understand course content at their own pace and according to their interests [8].

PBL fosters student-centered learning by encouraging collaboration in solving real-world problems [9]. Educational models have shifted from teacher-centered to student-centered learning over the past few decades; the teacher’s role has evolved from instructing to facilitating self-directed learning, fostering an effective learning environment [10]. Studies indicate that PBL strengthens students’ interest and motivation by connecting their work to real-world applications [9,11].

Integrating flipped classrooms with PBL establishes a robust educational model that combines the strengths of both approaches. This hybrid method encourages students to better understand theory and practice, fostering comprehensive knowledge and skills [7,12]. This integrated approach addresses the limitations of traditional methods, particularly regarding older adult health education, where practical experience and teamwork skills are typically lacking [13,14].

However, while flipped classrooms and PBL have successfully improved learning outcomes across various fields, research examining their combined effects on students’ willingness to serve older adults, teamwork skills, and overall learning outcomes is limited, particularly in the context of older adult health education. Recent studies have explored the impact of these methods on educational outcomes such as subject mastery, collaboration, and self-directed learning [8,9]. However, research on how these methods affect older adult health education remains limited.

Considering the positive results of flipped classrooms and PBL in other fields, further research is needed to assess their specific impact on older adult health education. As such, this study evaluates the effect of integrating flipped classrooms with PBL on enhancing students’ willingness to serve older adults, teamwork skills, and overall learning outcomes in older adult health education.

## 2. Methods

### 2.1. Study Design

This study employed a one-group pretest–posttest quasi-experimental mixed-methods design to evaluate the effectiveness of integrating flipped classrooms and PBL into an experiential health internship course focusing on older adult care. This study was conducted at a university in central Taiwan, and the participants included 88 interdisciplinary students from various departments such as nursing, medical sociology, public health, and psychology. This two-credit course spanned over 18 weeks and was held from August 2023 to July 2024.

This study combined both quantitative and qualitative data collection and analysis. Quantitative data were gathered using standardized scales administered before and after the course, while qualitative data were obtained from students’ reflective journals, allowing for a richer interpretation of learning experiences. This mixed-methods approach provided a more comprehensive understanding of the educational impact.

### 2.2. Participant Selection Criteria

Inclusion criteria for participant recruitment were as follows: (1) students who had not previously taken the older adult health experiential internship course; (2) students who voluntarily participated in the study after being informed of its purpose.

Exclusion criteria included students who had previously taken the course. Among the 90 students initially enrolled, two were classified as loss to follow-up (one due to course withdrawal and the other due to excessive absences), resulting in a final sample size of 88 participants. The enrollment rate was 97.8%.

### 2.3. Intervention

The teaching intervention combined the flipped classroom approach with PBL and was implemented over an 18-week course. The course was structured as follows:

#### 2.3.1. Weeks 1 to 6: Foundational Learning

During the foundational learning phase, students primarily focused on theoretical learning, including reviewing digital materials and watching video lectures on older adult care. Course content includes comprehensive geriatric assessment (CGA), communication techniques with older adults, and clinical skills (e.g., turning, percussion, wheelchair transfers, nasogastric tube feeding, and vital sign monitoring). Students were introduced to the basic concepts and planning in designing activities for older adults.

Students were expected to dedicate approximately 2 h per week to foundational learning, including digital module review and online lecture engagement. In addition, the instructors introduced the principles of PBL and facilitated classroom discussions and exercises to help students planning their elderly care projects, laying the foundation for the PBL tasks conducted in later weeks.

#### 2.3.2. Weeks 7 to 17: Clinical Practicum and Activity Design

##### Clinical Practicum

All students were assigned to a single long-term care facility and organized into 12 groups, each consisting of seven to eight members. These groups rotated through different units to gain exposure to diverse care settings. Students practiced essential long-term care skills through bedside teaching, including monitoring vital signs, feeding, and performing CGA. Each group received approximately two hours per week of onsite guidance. The instruction was provided collaboratively by two university faculty members and designated clinical mentors, including three registered nurses and nine senior caregivers. Each instructor oversaw six groups, totaling about 44 students. Tutoring strategies included bedside instruction, small group discussions, real-time feedback, post-care debriefing sessions, and reflective journal reviews. Activity design project and PBL implementation students were required to complete two major tasks. First, each of the 12 groups submitted a written CGA report. Second, they formed six larger teams (14–15 members per team) to plan and conduct six tailored activity sessions for older adults. The activity project followed a semi-guided approach. Instructors delivered essential theoretical instruction during weeks 1 to 6, while students developed activity plans independently based on the assessed needs of the residents.

From weeks 7 to 17, each team conducted a 30-min rehearsal, a 1-h activity session, and a 30-min feedback meeting with instructors and social workers.

Both faculty members completed eight hours of certified PBL training, ensuring alignment between instructional practice and the principles of problem-based learning.

#### 2.3.3. Week 18: Presentation and Reflection

In the final week, students submitted three types of final deliverables: (1) a group-written CGA report, (2) a group activity design summary, and (3) individual reflection reports and peer evaluations. Students delivered oral presentations using PowerPoint slides to showcase their project development, implementation outcomes, and personal growth throughout the practicum. These sessions were attended by instructors, social workers, and clinical mentors, who offered formative feedback and guided reflection. The reflective process was further supported using written self-reflections, encouraging students to analyze their clinical experience, identify challenges encountered, and articulate learning gains in professional practice and teamwork.

### 2.4. Measurement

#### 2.4.1. Demographic Information Form

The demographic information form collected data on students’ age, gender, major, living situation during the 6 months prior to the course, family structure, recent (within six months/six months prior) interactions with grandparents, frequency of contact with older adults over 65 years old, previous courses related to older adult care, related work experience, and level of concern regarding older adult issues.

#### 2.4.2. Teamwork Skill Scale

The Teamwork Skill Scale [15] comprises 16 items across four dimensions: communication (6 items), coordination (3 items), effort (3 items), and cohesion (4 items). Scores are rated on a five-point Likert scale (1 = strongly disagree; 5 = strongly agree), with total scores ranging from 16 to 80. Higher scores indicate better teamwork abilities. The scale demonstrated strong internal consistency, with a Cronbach’s alpha of 0.95. Cronbach’s alpha in this study achieved 0.97, with subscale reliabilities as follows: communication (0.96), coordination (0.85), effort (0.93), and cohesion (0.92).

#### 2.4.3. Willingness to Serve the Older Adults Scale

The Willingness to Serve the Older Adults Scale [16] comprises 17 items across two dimensions: helping and caregiving (11 items) and approaching and serving (6 items). Scores are rated on a five-point Likert scale from 1 (strongly disagree) to 5 (strongly agree), with a total score ranging from 17 to 85. The overall scale in the present study had a Cronbach’s alpha of 0.95, with subscale reliabilities of 0.93 for helping and caregiving and 0.87 for approaching and serving.

#### 2.4.4. Learning Outcomes Scale

Adapted from Kuo’s [17] Student Satisfaction with Off-campus Internship Scale, the learning outcomes scale in this study comprises 22 items across four subscales: learning growth (10 items), teacher guidance (4 items), internship unit (5 items), and personal factors (3 items). Scored on a five-point Likert scale, total scores range from 22 to 110. Higher scores indicate greater learning effectiveness. The scale was validated by three experts in long-term care and related fields, with a content validity index (CVI) of 0.94. The overall scale’s Cronbach’s alpha in this study was 0.97, with subscale reliabilities as follows: learning growth (0.96), teacher guidance (0.93), internship unit (0.95), and personal factors (0.95).

### 2.5. Statistical Procedures

Data were analyzed using SPSS for Windows (version 25.0, IBM Corp., Armonk, NY, USA). This study used descriptive statistics to summarize the data, including frequency distributions, percentages, means, and standard deviations. Paired t-tests were used to compare pre- and post-intervention scores on students’ willingness to serve older adults, teamwork skills, and learning effectiveness. Independent sample t-tests, Pearson’s correlations, and one-way ANOVA were employed to explore the relationships between demographic variables and the study’s outcomes. For missing data, mean substitution was used, replacing missing values with the mean of the respective variable to ensure data completeness and minimize sample loss.

### 2.6. Data Collection

Before the course began, this study was approved by the Human Research Ethics Committee of a hospital in central Taiwan. The researcher obtained formal permission from the management of a regional nursing home to allow student participation during the internship through an official letter issued by the university. The purpose and significance of the study were explained to all students prior to data collection, and written informed consent was obtained. This study employed a mixed-methods design, integrating both quantitative and qualitative data to provide a more comprehensive evaluation of the intervention’s impact. The pretest phase involved structured questionnaires administered prior to the commencement of the 18-week course. These questionnaires assessed demographic characteristics, willingness to serve older adults, teamwork abilities, and self-perceived learning outcomes. Students were also encouraged to engage in reflective thinking throughout the duration of the course. Following the intervention, posttest questionnaires were distributed to measure the same variables. At the end of the semester, each group submitted a comprehensive project report and group assessment. Additionally, students completed individual written reflections, self-assessments, and peer evaluations, all of which were submitted via the e-Class digital learning platform. Beyond the quantitative survey data, all 88 students submitted individual reflection reports at the conclusion of the course using the e-Class platform. These narratives yielded rich qualitative insights into students’ subjective learning experiences, emotional engagement, and perceived professional growth throughout the course and practicum. A thematic analysis was independently conducted by two researchers to identify recurring patterns and core themes, such as the development of empathy, enhanced understanding of geriatric care, and increased intrinsic motivation for learning.

Although the original study design approved by the IRB was primarily quantitative in nature, the reflective journals collected as part of routine course activities provided valuable qualitative insights. These qualitative data were derived from existing curricular assignments and did not involve any additional intervention or impose further burden on students. Therefore, their inclusion remained fully consistent with the scope and ethical principles approved by the IRB. These qualitative findings offered a deeper understanding of the mechanisms through which the educational intervention exerted its effects. Their integration into the unified study design enhanced the depth and comprehensiveness of the evaluation framework.

### 2.7. Ethical Considerations

This study was approved by the Institutional Review Board (IRB No. CS1-23XX8) of a hospital in Taichung. All participants signed informed consent forms. Data collection strictly adhered to ethical standards, ensuring participant confidentiality and the right to withdraw from the study without affecting their educational opportunities.

## 3. Results

Among the 90 students initially participating in the course, two were classified as lost to follow-up, with one due to course withdrawal and the other due to excessive absences, resulting in a final sample size of 88 participants. The retention rate was 97.8%.

Table 1 presents the demographic characteristics of the 88 students participating in the course. The average age of the participants was 22.34 years (SD = 1.37), and the majority were female (76.1%). Most students were enrolled in the nursing program (64.8%), while others were from medical sociology (23.7%) or other health-related majors (8.0%). Regarding living arrangements, 55.7% of the students rented accommodations, 33.0% lived with family, and 11.4% resided in dormitories or with relatives or friends. Moreover, 63.6% of the students had previously taken courses related to older adult care issues, and 71.6% had work experience related to elder care.

Table 2 shows the differences in students’ willingness to serve older adults, teamwork skills, and learning outcomes before and after the intervention. The results indicate significant improvements across all domains.

Willingness to Serve Older Adults: The average score increased from 68.93 (SD = 10.05) to 73.15 (SD = 9.93) (*t* = 4.17, *p* < 0.001). Significant improvements were observed in the “help and care” and “approach and care” subscales, particularly regarding students’ willingness to assist with tasks such as mailing letters, recognizing personal responsibility in helping and serving older adults, and providing support with personal hygiene. Areas with minor improvements included communication between team members, spontaneous use of various communication channels, and a stronger sense of group belonging.

Teamwork Skills: Teamwork scores increased from 67.33 (SD = 9.61) to 71.45 (SD = 8.88) (*t* = 3.68, *p* < 0.001). Positive changes were observed in subcategories such as communication, coordination, effort, and cohesion. The most significant improvements were seen in students’ pride in being part of the team, ability to coordinate tasks, and prioritization of classroom reports.

Learning Outcomes: Learning outcomes increased significantly from 89.84 (SD = 11.83) to 102.14 (SD = 8.91) (*t* = 74.26, *p* < 0.001). All indicators, including learning growth, teacher guidance, internship experience, and personal factors, showed significant improvements. The most significant changes were in areas such as the comprehensive orientation provided during internships, the quality of the internship work environment, and timely encouragement or guidance from the head nurse, caregiver, or social worker.

In addition to the improvements in individual dimensions as shown in Table 2, the final evaluation of the course included both individual and group performance. The final grades were composed of five components: group-based CGA reports (25%), group activity design projects (25%), attendance (20%), individual reflection reports (20%), and peer/self-evaluation (10%). Group scores for CGA ranged from 88 to 95, and activity design scores ranged from 90 to 95. Individual scores ranged from 80 to 95. Based on the weighted grading scheme, the overall final grade distribution was as follows: A (90–100), 45 students (51.1%); B+ (85–89), 31 students (35.2%); B (80–84), 12 students (13.6%). No student scored below 80. These results indicate that the majority of students achieved high academic performance, demonstrating not only strong individual competence but also effective teamwork during the course.

Table 3 presents the differences in students’ willingness to serve older adults, teamwork skills, and learning outcomes based on demographic characteristics. Regarding willingness to serve older adults, students from extended/modified extended families, those with close or very close recent (or past) interactions with grandparents, and those with frequent or very frequent past interactions with grandparents had higher willingness scores. Additionally, students who expressed greater concern for older adult issues also demonstrated significantly higher willingness scores. Regarding teamwork skills, students majoring in nursing and those who had lived in school dormitories or with relatives in the past six months demonstrated better teamwork skills. The findings indicate that younger students, those majoring in nursing, and those who had lived in school dormitories or with relatives within the past six months achieved significantly higher posttest learning outcome scores than others (*p* < 0.05) (Table 3).

### 3.1. Qualitative Findings

To enhance the interpretation of the quantitative results and provide a more comprehensive understanding of the impact of the educational intervention, a thematic analysis was conducted on reflective journals submitted by 88 students at the end of the course.

The analysis identified three overarching themes and six subthemes (see Table 4) that illustrate how the integration of flipped classrooms and PBL facilitated students’ emotional engagement, strengthened collaborative learning through peer reflection, and supported the application of knowledge through clinical experiences.

These qualitative insights underscore the importance of a mixed-methods approach in experiential learning of older adult health education.

### 3.2. Theme 1: Emotional Engagement Through Interaction with Older Adults

Many students reported that direct interaction with older adults significantly altered their previous stereotypes and misconceptions. A total of 53 students (60.23%) described developing emotional resonance and a deeper understanding during these interactions. One student wrote, “While chatting with an older male in the care facility, he said, ‘You are the first student who actually sat down and listened to me.’ That moment was deeply moving and made me realize how much older adults long to be heard and understood.” Such first-hand experiences appeared to promote empathy and a greater sense of social responsibility. Furthermore, 45 students (51.14%) stated that participating in activity design and companionship initiatives enhanced their motivation to serve older adults. As one student expressed, “I was initially afraid, but now I genuinely look forward to talking with older adults again, because I realize that my presence means something to them.”

### 3.3. Theme 2: Enhanced Learning Through Teamwork and Peer Reflection

Group collaboration and PBL were frequently identified by students as key factors in promoting learning engagement and interpersonal development. A total of 39 students (44.32%) reported that group projects helped them improve their communication and negotiation skills. One student reflected, “I used to interrupt others all the time, but this time I learned to listen first, which made the whole team work more smoothly.” Additionally, 28 students (31.82%) noted that although conflicts emerged during teamwork, they gained important experience in respectful communication and conflict resolution. A student remarked, “Although we had disagreements at first, by the end we became a really great team.” Moreover, 26 students (29.55%) described a strong sense of accomplishment after completing their team projects, which contributed to increased motivation and a deeper sense of responsibility towards their learning.

### 3.4. Theme 3: Application of Knowledge and Learning Outcomes Through Clinical Practice

The majority of students indicated that hands-on experience in long term care settings allowed them to meaningfully apply classroom knowledge in real life situations. Specifically, 51 students (57.95%) reported that participating in caregiving tasks such as feeding, transferring patients, and monitoring vital signs boosted their confidence in practical skills and reinforced their sense of professional identity. As one student reflected, “At first I was really nervous about feeding an older adult, but after doing it once, I realized I was capable and even did a good job. That experience gave me confidence in my future as a caregiver.” Additionally, 46 students (52.27%) shared that the clinical practicum was instrumental in helping them bridge theory and practice. One student noted, “Before, I just memorized everything from textbooks, but now I actually know how to apply those assessment techniques in clinical settings.”

## 4. Discussion

### 4.1. Impact of Integrating Flipped Classroom and Project-Based Learning (PBL) on Students’ Willingness to Serve Older Adults, Teamwork Skills, and Overall Learning Outcomes

This study found that integrating a flipped classroom approach and PBL significantly improved students’ willingness to serve older adults, teamwork skills, and learning outcomes. Specifically, willingness to serve older adults increased from 68.93 to 73.15, teamwork skills from 67.33 to 71.45, and learning outcomes from 89.84 to 102.14. These findings highlight the positive impact of innovative teaching methods on student engagement and learning effectiveness [6,18].

Regarding willingness to serve older adults, the most significant improvements were seen in practical assistance tasks, such as mailing letters and supporting personal hygiene, reflecting an enhanced sense of responsibility among students [2]. The integrated flipped classroom and PBL approach encouraged students to engage more actively in older adult care tasks in their daily lives [19].

The qualitative data of this study further substantiated the quantitative findings. Observations revealed that students formed emotional bonds with older adults through direct interaction, which helped challenge their existing stereotypes and enhanced their willingness to serve [6]. Approximately 60% of the students reported a growth in empathy and social responsibility after participating in activity design and companionship programs with older adults. These findings are consistent with Llach and Bastida [2], suggesting that such transformation may stem from extended and meaningful participation, which shifts students from passive learners to active caregivers. Moreover, PBL strategies are known to enhance students’ motivation for social engagement [9,18].

Teamwork skills showed marked improvements, particularly in coordination (from 12.81 to 13.76) and effort (from 12.15 to 13.09). This finding aligns with past research indicating that PBL strengthens collaboration and task distribution among teams [9]. However, communication skills improved to a lesser extent, particularly in items such as “spontaneous use of various communication channels” and “communication between team members.” These findings suggest that students’ communication skills still need further improvement, while teachers could introduce more diverse communication channels [20]. Furthermore, course designs could incorporate more interactive opportunities, e.g., role-playing, simulations, and group discussions, to advance communication skills within teams [21,22].

The thematic analysis also indicated that teamwork and peer reflection significantly enhanced students’ communication and coordination abilities. Approximately one-third of the students reported noticeable improvements in conflict resolution and active listening skills. These developments were likely attributed to the group-based activity design integrated into the course, which required students to engage in collaborative decision-making and shared responsibility [10]. It is recommended that future course designs continue to incorporate structured role-playing and simulation-based learning scenarios to deepen student engagement and foster better team interaction skills [21,22].

Regarding learning outcomes, the greatest improvement was seen in learning growth (from 38.60 to 46.10), followed by internship experience, personal factors, and teacher guidance. The integrated flipped classroom and PBL approach significantly improved students’ ability to translate theoretical knowledge into real-world caregiving practice, particularly during clinical internships and elder care activities [23]. In addition, students’ reflections echoed the findings of the quantitative data. More than half of the students indicated that participating in hands-on caregiving tasks, such as feeding older adults and monitoring vital signs, significantly enhanced their clinical confidence and sense of professional identity. These authentic experiences enabled students to effectively transfer and apply theoretical knowledge acquired in the classroom to real-world clinical contexts. This highlights the essential role of experiential learning in geriatric care education [23]. It is recommended that future experiential practicum courses incorporate interdisciplinary collaboration and practice-oriented problem-solving tasks, such as interprofessional team assessments, the development of care plans for older adults, and simulated home visit scenarios, in order to enhance students’ learning motivation and strengthen their ability to apply classroom knowledge in actual care environments [8,24]. By engaging students in real-world problem solving, these methods stimulated their interest and motivation, leading to significant growth in learning outcomes [7,9]. The findings also indicate that students who received comprehensive introductions to internship units, a supportive working environment, and encouragement and guidance from head nurses, caregivers, and social workers demonstrated better learning outcomes. The relevant literature supports the finding that thorough introductions from internship units help students adapt to new environments more quickly while improving their confidence and sense of security [11,24]. A working environment facilitating hands-on interaction with older adults enables students to apply theoretical knowledge in practical contexts, enhancing their practical skills and career readiness [20,24]. Moreover, this study found that hands-on clinical practicum experiences played a pivotal role in shaping students’ learning outcomes and attitudinal transformation. Consistent with the findings of Carlsson et al. [24] and Chikeme et al. [7], students who engaged in practical caregiving tasks such as feeding, transferring patients, and monitoring vital signs in long-term care settings not only enhanced their clinical confidence and sense of professional identity but also demonstrated a greater ability to translate theoretical classroom knowledge into authentic real-world applications.

### 4.2. Impact of Demographic Characteristics on Students’ Willingness to Serve Older Adults, Teamwork Skills, and Learning Outcomes

This study’s findings imply that students from extended or blended families were significantly more willing to serve older adults than those from single-parent or skipped-generation families. As these family structures typically involve multigenerational living arrangements, research indicates that students from extended or blended families may have more frequent contact with older adults [25]. An extended family environment enables students to become accustomed to living with and interacting with older adults from a young age. This environment can lead to a deeper understanding of the needs and care of older adults [26]. Early exposure and experience may help students be more inclined to engage in older adult healthcare courses while becoming more willing to serve older adults [27]. In contrast, students from single-parent or skipped-generation families may lack experience living with older adults, which could affect their willingness to serve older adults [13]. Moreover, this study found that students with close or very close interactions with grandparents during the past six months, as well as those with frequent or very frequent past interactions with grandparents, showed significantly higher willingness to serve older adults than those with less frequent interactions or those considered less close. Research indicates that students who have close relationships with their grandparents develop deep emotional bonds and feelings of care through these interactions [18]. These emotional connections may stimulate their concern and responsibility for the well-being of older adults, making them more willing to participate in older adult care services [27]. Furthermore, students who frequently interact with their grandparents have more opportunities to observe while participating in the daily lives of older adults. This helps them better understand their needs more effectively, increasing their willingness to serve older adults during courses and internships [23]. As such, teachers can add multigenerational learning environments in their courses, providing students with more opportunities to interact with people from different age groups, particularly with older adults, through community activities, volunteer services, or off-campus visits, enhancing students’ willingness to serve older people [2].

This study also found that nursing students showed significantly better teamwork skills than students majoring in medical sociology. Students who lived in school dormitories or with relatives within the last six months also demonstrated significantly better teamwork skills than those who lived at home. Superior teamwork skills of nursing students compared to medical sociology students may be related to the frequent participation in group activities, role-playing, and clinical simulation training in nursing education that requires close collaboration among students. These practical experiences improve students’ professional skills while enhancing teamwork [13,23]. Moreover, this study found that students who had lived in school dormitories or with relatives within the past six months had significantly better teamwork skills and learning outcomes than those living at home. This could be because students living in school dormitories often share living spaces with peers, and to communicate and collaborate with others more often [18]. Students residing with relatives may also enjoy higher levels of social support and emotional care, helping them seek help and resources more readily when facing challenges [2]. In contrast, students living at home may rely more on family support while lacking opportunities for independent living and collaboration with peers, potentially limiting their emphasis on teamwork and learning [21].

The findings from this study also indicate that younger students had better learning outcomes, aligning with the findings of Yue [28]. This may be because younger university students are more adaptable to new teaching methods or more interested in new technologies and learning models [7]. In this study, nursing and medical sociology students showed better learning outcomes, possibly because they possess foundational knowledge and practical experience related to healthcare. This could have enabled them to adapt and interact more effectively with older adults during the older adult experiential health internship course, resulting in better learning outcomes [13].

The study limitations are as follows. First, we used a one-group pretest–posttest design without a control group, which may have led to external influencing factors. The lack of a control group is a commonly recognized limitation in educational intervention research, particularly in real-world classroom settings where ethical and logistical constraints often make random assignment impractical. To attenuate this limitation, future research may consider using quasi-experimental designs involving non-equivalent comparison groups, such as students enrolled in similar courses taught using traditional methods. It may also be feasible to establish internal control groups within the same university by comparing students from different academic years or departments, using matched sampling strategies to ensure baseline equivalence. Second, the data relied primarily on students’ self-reports, which may be subject to several biases including social desirability bias, recall bias, and response-set bias. Students might have unintentionally overreported their learning gains or willingness to serve due to the positive nature of the course content and teacher expectations. Future research should combine observations, teacher evaluations, or peer assessments to mitigate these limitations and enhance data credibility. Third, the study sample comprised students from one university, which may result in sample homogeneity and limit the generalizability of the findings. The cultural, academic, and institutional context of a single university might not represent the experiences or outcomes of students in other regions or programs. Fourth, although the clinical practicum constituted a key experiential learning component, its individual contribution was not disentangled from that of the flipped classroom and PBL strategies. This limitation may confound interpretations regarding the intervention’s overall effectiveness. Future studies are encouraged to employ factorial or comparative designs to better distinguish the effects of each component.

Future research should investigate the long-term learning impacts of the integrated flipped classroom and PBL in terms of academic achievement, career development, problem-solving abilities, and teamwork skills. Researchers should also consider the application of this teaching model in other healthcare fields, such as mental health, to further validate its generalizability. Moreover, supporting and training educators is crucial, as teacher competence directly influences the successful implementation of these innovative approaches.

## 5. Conclusions

This study confirmed the significant benefits of integrating a flipped classroom approach with PBL in older adult health education. These innovative pedagogical methods substantially enhanced students’ willingness to serve older adults, improved teamwork competencies, and elevated overall learning outcomes. The flipped classroom model, which introduces theoretical content prior to class and dedicates in-class time to interactive application, was found to increase student engagement and deepen understanding. PBL, on the other hand, fostered critical thinking and collaboration by encouraging students to address real-world caregiving challenges.

More importantly, this study found that the integration of flipped classrooms and PBL not only supported active and reflective learning but also enabled students to effectively transfer theoretical knowledge into real-life contexts. Both quantitative and qualitative findings underscored improvements in students’ empathy, communication, professional confidence, and ability to work collaboratively. Students’ emotional connections with older adults, formed during activity design and caregiving tasks, further reinforced their motivation to serve and their understanding of holistic care.

In particular, the clinical practicum component played a pivotal role in helping students bridging classroom learning with hands-on caregiving. Through experiences such as feeding, patient transfer, and vital sign monitoring, students reported increased confidence and professional identity. These findings highlight the importance of integrating structured, real-world clinical experiences to amplify the effectiveness of flipped classroom and PBL strategies. This synergy between theory and practice should be considered a foundational pillar in future curriculum design for geriatric care education.

In conclusion, the combined application of flipped classrooms and PBL presents a promising and scalable educational model for enhancing health education in aging societies. By fostering a more engaging, practical, and interactive learning environment, this approach better prepares students for real-world challenges in long-term care settings. These findings lay a foundation for future educational reforms and emphasize the need for ongoing support and training for educators to effectively implement these innovative teaching practices.

## Figures and Tables

**Table 1:** Demographic characteristics of students in the older adult experiential health internship course (*N* = 88).

Variables	All	
	*N*	%
Age Mean (SD)	22.34 (1.37)	
Gender		
Female	67	76.1
Male	21	23.9
Academic major		
Nursing	57	64.8
Medical sociology	24	23.7
Nutrition/psychology/physical therapy /public health/medical imaging	7	8.0
Current (within 6 months) living situation		
Dormitory/relative’s home	10	11.4
Renting	49	55.7
Family home	29	33.0
Living situation during the 6 months prior to the course		
Dormitory/relative’s home	16	18.2
Renting	42	47.7
Family home	30	34.1
Religious beliefs		
No	55	62.5
Yes	33	37.5
Family structure		
Extended/blended family	21	23.9
Nuclear family	54	61.4
Single-parent/skipped-generation family	13	14.8
Interactions with grandparents during the 6 months prior to the course		
Very not close/not close	17	19.3
Neutral	35	39.8
Close/very close	36	40.9
Previous (6 months prior) interaction with grandparents		
Very not close/not close	16	18.2
Neutral	38	43.2
Close/very close	34	38.6
Recent frequency of contact with grandparents		
Very infrequent/infrequent	28	31.8
Neutral	36	40.9
Frequent/very frequent	24	27.3
Previous frequency of contact with grandparents		
Very infrequent/infrequent	2	26.1
Neutral	39	44.3
Frequent/very frequent	26	29.5
Frequency of contact with older adults (65+ years)		
Very infrequent/infrequent	28	31.8
Neutral	30	34.1
Frequent/very frequent	30	34.1
Completed courses on older adult issues		
No	32	36.4
Yes	56	63.6
Work experience in older adult care		
None	25	28.4
Very little	18	20.5
Little	24	27.3
A lot	21	23.9
Concern for older adult issues		
Unconcerned/neutral	48	54.5
Concerned	36	40.9
Very concerned	4	4.5

Note: Mean (M), Standard deviation (SD).

**Table 2:** Paired sample *t*-test analysis of students’ willingness to serve the older adults, teamwork, and learning outcomes before and after the PBL integration with the flipped classroom in the older adult experiential health internship course (*N* = 88).

Variable	Pretest		Posttest		95% CI	*t*	*p*
	Mean Score	SD	Mean Score	SD			
Willingness to serve older adults (17–85)	68.93	10.05	73.15	9.93	[2.21~6.22]	4.17	0.001 ***
Help and care (11–55)	41.47	5.82	48.36	6.15	[5.62~8.18]	10.70	<0.0001 ***
Approach and care (6–30)	23.25	3.96	24.78	4.17	[0.75~2.32]	3.87	<0.001 ***
Teamwork (16–80)	67.33	9.61	71.45	8.88	[1.89~6.36]	3.68	<0.001 ***
Communication (6–30)	25.72	3.73	27.09	3.23	[0.47~2.28]	3.03	<0.01 **
Coordination (3–15)	12.81	1.76	13.73	1.61	[0.48~1.36]	4.20	<0.0001 ***
Effort (3–15)	12.15	2.25	13.09	2.09	[0.42~1.47]	3.58	<0.001 ***
Cohesion (4–20)	16.62	2.79	17.55	2.70	[0.25~1.52]	2.79	<0.01 **
Learning outcomes (22–110)	89.84	11.83	102.14	8.91	[61.83~65.24]	74.26	<0.001 ***
Learning growth (10–50)	38.60	5.91	46.10	4.70	[6.38~8.62]	13.33	<0.001 ***
Teacher guidance (4–20)	17.42	2.36	18.60	1.99	[0.66~1.71]	4.47	<0.001 ***
Internship unit (5–25)	21.01	3.19	23.47	2.06	[1.77~3.13]	7.18	<0.001 ***
Personal factors (3–15)	12.81	1.93	13.97	1.44	[0.78~1.54]	6.04	<0.001 ***

Note: ** *p* < 0.01, *** *p* < 0.001.

**Table 3:** Differences in students’ demographic characteristics, willingness to serve older adults, teamwork skills, and learning outcomes (*N* = 88).

Variables	Willingness to Serve Older Adults		Teamwork		Learning Outcomes	
	M (SD)	t/F/p	M (SD)	t/F/p	M (SD)	t/F/p
Age ^a^	68.93 (10.05)	−0.05	67.33 (9.61)	−0.16	89.84 (11.83)	−0.27 *
Academic major ^c^		1.13		10.37 ***		3.36 *
Scheffé test			Nursing > Medical sociology		Nursing > Medical sociology	
Living situation during the 6 months prior to the course ^c^		2.23		3.55 *		3.83 *
Scheffé test			Dormitory /relative’s home > Family home		Dormitory /relative’s home > Family home	
Family structure ^c^		5.54 **		0.90		1.56
Scheffé test	Extended/blended family > Single-parent/skipped-genertion family					
Recent (within 6 months) interaction with grandparents ^c^		4.73 *		0.35		1.81
Scheffé test	Very not close/not close < Close/very close					
Previous (more than 6 months ago) interaction with grandparents ^c^		3.69 *		1.38		0.66
Scheffé test	Close/very close > Very not close /not close					
Previous frequency of contact with grandparents ^c^		4.58 *				
Scheffé test	Neutral > Very infrequent/infrequent					
Concern for older adult issues ^c^		4.71 *		0.24		0.72
Scheffé test	Very concerned > Unconcerned/neutral					

Note: Only variables showing statistically significant differences and Scheffé post hoc results are presented. * *p* < 0.05, ** *p* < 0.01, *** *p* < 0.001; ^a^ Pearson’s correlation coefficient; ^c^ Analysis of variance (ANOVA).

**Table 4:** Thematic analysis of reflective journals (*N* = 88).

Main Theme	Sub-Theme	*N*	%
1. Emotional Engagement through Interaction with Older Adults	1.1 Changed stereotypes and developed empathy	53	60.23%
	1.2 Increased motivation to serve older adults	45	51.14%
2. Strengthened Learning through Teamwork and Peer Reflection	2.1 Improved communication and negotiation skills	39	44.32%
	2.2 Constructive conflict resolution and mutual respect	28	31.82%
	2.3 Sense of accomplishment from group project	26	29.55%
3. Application of Knowledge and Learning Outcomes through Clinical Practice	3.1 Enhanced confidence and professional identity in clinical practice	51	57.95%
	3.2 Integration of theory and clinical practice	46	52.27%

## Data Availability

The data supporting this study’s findings are available on request from the corresponding author. Data are not publicly available due to privacy and ethical restrictions.

## Public Involvement Statement

No public involvement in any aspect of this research.

## Guidelines and Standards Statement

This manuscript was prepared in accordance with the TREND (Transparent Reporting of Evaluations with Nonrandomized Designs) Statement [29]. For further details, please refer to: https://ajph.aphapublications.org/doi/full/10.2105/AJPH.94.3.361/ (accessed on 20 July 2025).

## Abbreviations

PBL	Project-Based Learning
SD	Standard Deviation
CVI	Content Validity Index
IRB	Institutional Review Board
